# Reconstructing fatherhood after childhood trauma: a phenomenological study of Turkish men

**DOI:** 10.3389/fpsyg.2026.1886488

**Published:** 2026-07-21

**Authors:** Özge Erduran Tekin, Eda Ermagan Caglar, Cagla Sahin

**Affiliations:** 1 Department of Educational Sciences, Turkish Air Force Academy, National Defense University, İstanbul, Türkiye; 2 Department of Psychology, Faculty of Humanities and Social Sciences, Sakarya University, Sakarya, Türkiye; 3 Instıtute of Social Sciences, Uskudar University, İstanbul, Türkiye

**Keywords:** childhood trauma, fatherhood, intergenerational trauma, interpretative phenomenological analysis, masculinity norms, parenting experiences

## Abstract

Childhood trauma can shape emotional development and relationships across the lifespan, yet fathers’ subjective experiences remain underexplored. This qualitative study examined how Turkish fathers with childhood trauma histories experience fatherhood and everyday parenting. Using interpretative phenomenological analysis, 17 fathers with elevated Childhood Trauma Questionnaire scores participated in semi-structured online interviews. Six themes emerged: emotional wounds, masculine norms, authority and closeness in parenting, emotional triggering, transformation, and changing fatherhood experiences. Fatherhood often reactivated unresolved childhood experiences, especially during stressful interactions with children. Traditional masculinity norms limited emotional expression and help-seeking. Participants expressed a strong desire to parent differently from their own fathers and prevent harmful intergenerational patterns. For some, fatherhood became a turning point that encouraged self-reflection, emotional growth, and more intentional parenting. The findings suggest that fatherhood may simultaneously reactivate childhood trauma and create opportunities for personal change and healthier parenting practices.

## Introduction

Childhood trauma is widely regarded as one of the most psychologically damaging forms of adversity because it occurs during a developmentally vulnerable period and often involves neglect or abuse perpetrated by caregivers (Herman, 1992). In the present study, childhood trauma is conceptualized as caregiver-related maltreatment occurring during developmentally sensitive periods, encompassing the five forms assessed by the Childhood Trauma Questionnaire: emotional, physical, and sexual abuse, as well as emotional and physical neglect (Bernstein et al., 1994). This operational definition deliberately centers experiences perpetrated or permitted within the caregiving relationship, rather than the broader spectrum of adverse childhood experiences (ACEs). Accordingly, while related forms of early adversity such as witnessing domestic violence, parental abandonment, or poverty-related hardship may co-occur with and compound maltreatment, they fall within the scope of the present conceptualization only insofar as they are expressed through these maltreatment dimensions. This distinction matters because the terms *adversity* and *trauma* are often used interchangeably. However, they are not equivalent: not all adversity is traumatic, and not all trauma arises from identifiable adversity. The Adverse Childhood Experiences (ACEs) framework casts a deliberately wide net, encompassing household dysfunction alongside abuse and neglect (Felitti et al., 1998). In contrast, Developmental Trauma and Complex Trauma frameworks foreground the chronic, interpersonal, and caregiver-bound nature of harm and its cumulative impact on emotional regulation, attachment, and self-organization (Cook et al., 2005; van der Kolk, 2005; Herman, 1992). The present study is positioned primarily within this latter tradition; its focus is not the full breadth of childhood adversity but the relational maltreatment that the complex trauma literature identifies as particularly consequential for later interpersonal and caregiving functioning. Framing the phenomenon in these terms clarifies why the fathers in this study are understood not simply as having endured difficult childhoods but as carrying relationally embedded wounds that are likely to be reactivated within the caregiving relationships they later form. Experiences of childhood trauma are highly prevalent worldwide and can negatively affect individuals’ physical, emotional, and psychological development (Petruccelli et al., 2019). A growing body of research indicates that the consequences of childhood trauma are not limited to the period in which they occur but may also lead to significant psychological outcomes later in life (Hales et al., 2023). Individuals who have experienced childhood trauma may develop a range of psychological reactions, including identity disturbances, diminished self-esteem, intense feelings of guilt and shame, and anger toward those responsible for the traumatic experiences (Helvacı Çelik and Hocaoğlu, 2018; Zhang et al., 2025).

Moreover, childhood trauma has been strongly associated with a variety of psychopathological outcomes in adulthood, including depression, anxiety disorders, substance use problems, physical health difficulties, and functional impairments (Copeland et al., 2018; Perry and Szalavitz, 2018). In recent years, the literature has increasingly emphasized that the impact of childhood trauma extends beyond individuals’ mental health and may also influence parenting behaviors and the developmental trajectories of subsequent generations. In particular, research has shown that parents’ childhood trauma is associated with higher parenting stress, reduced parental sensitivity, and lower-quality parent–child interactions (Racine et al., 2025; Rowell and Neal-Barnett, 2022). These findings suggest that trauma should not be viewed solely as an individual experience but rather as a process that may be transmitted across generations within family systems. At the same time, an exclusive focus on psychopathological outcomes risks producing a deficit-oriented account that obscures an equally robust body of evidence. A substantial literature documents that many individuals exposed to childhood trauma do not go on to replicate harm and instead demonstrate resilience, recovery, and in some cases, post-traumatic growth, with protective factors and supportive relationships moderating the long-term impact of early adversity (Bonanno, 2004; Masten, 2001). Within the parenting domain specifically, a history of childhood trauma does not determine caregiving outcomes. A considerable proportion of trauma-exposed parents become sensitive, reflective, and nurturing caregivers, and some appear to draw on their own histories as a source of motivation to parent differently (Fuchs et al., 2015; Shafer and Easton, 2021). Recognizing this heterogeneity is not merely a matter of balance; it is analytically necessary, because the conditions under which trauma is reproduced rather than interrupted are precisely what a study of fatherhood after childhood trauma must illuminate. The present study therefore adopts a deliberately non-deterministic stance, treating fatherhood as a context in which both the reactivation and the renegotiation of early experiences become possible.

### Fatherhood and childhood trauma

Parenthood represents a major developmental transition in adult life and entails psychological, social, and economic responsibilities associated with raising a child. Even for parents without a history of trauma, this transition can sometimes be challenging (Berg-Nielsen et al., 2002). However, parenting experiences may be particularly complex and stressful for individuals who have experienced childhood trauma (Zvara et al., 2015). Studies suggest that parents with histories of childhood trauma often encounter difficulties in emotion regulation, which can subsequently shape their parenting behaviors. In some cases, this may manifest in harsh or authoritarian parenting practices, whereas in others it may result in neglectful parenting styles (Banyard et al., 2003; DiLillo and Damashek, 2003; Wark and Vis, 2018). Research has also demonstrated that childhood trauma may be transmitted across generations through parenting behaviors (Widom et al., 2015). Nevertheless, some parents with traumatic childhood experiences appear able to develop more reflective and intentional parenting strategies, thereby interrupting this cycle of transmission (Fuchs et al., 2015).

For many years, studies examining the relationship between childhood trauma and parenting primarily focused on mothers (Shafer and Easton, 2021; Thornberry et al., 2012). More recent research, however, has highlighted the significant influence of fathers on children’s developmental and psychological outcomes (Cabrera et al., 2018; Schoppe-Sullivan and Fagan, 2020). Some studies even suggest that paternal behaviors may exert stronger effects on children’s emotional and behavioral adjustment than maternal behaviors in certain contexts (Flouri and Buchanan, 2003). Beyond the empirical observation that fathers have been comparatively neglected, however, there are substantive theoretical grounds for treating paternal trauma as a distinct object of inquiry rather than a variable to be added alongside maternal trauma. First, the pathways through which early adversity shapes caregiving appear to be partly gendered. Masculinity norms that discourage emotional disclosure, help-seeking, and the expression of vulnerability may channel the sequelae of childhood trauma into forms of paternal involvement and emotional availability that differ qualitatively from those documented among mothers (Connell and Messerschmidt, 2005; Kuscul and Adamsons, 2022). Second, fathers and mothers occupy distinct positions within culturally organized family systems, such that the same traumatic history may be elaborated through different role expectations, particularly in contexts where fatherhood is bound to authority and provision rather than to nurturance. Third, paternal and maternal influences on children’s emotional and behavioral development are not interchangeable, and in certain domains paternal behavior has been shown to exert independent or even stronger effects (Flouri and Buchanan, 2003). Taken together, these considerations indicate that the fatherhood experiences of men with childhood trauma cannot be adequately understood by extrapolating from research on mothers and instead require investigation in their own right. Research has further indicated that fathers with histories of childhood adversity tend to report greater psychological distress and more difficulties in fathering behaviors (Shafer and Easton, 2021). Similarly, some studies have found that men with histories of childhood trauma may show lower levels of paternal involvement during adulthood (Brown et al., 2020). Additionally, parents’ childhood trauma has been linked to emotional and behavioral outcomes in their children, suggesting the presence of intergenerational effects (Racine et al., 2025). More recent studies have also demonstrated that parental trauma histories may increase parenting stress (Bakhos et al., 2024), influence children’s attachment patterns, and shape early developmental processes among children whose fathers experienced adverse childhood experiences (Sirparanta et al., 2024). Furthermore, early-life adversity in men has been proposed to influence fathering behaviors through both biological and psychological mechanisms (Condon et al., 2022). Collectively, these findings indicate that childhood trauma may have significant implications for men’s experiences of fatherhood.

Fatherhood itself is a multifaceted phenomenon that is shaped by personal circumstances, motivations, and broader life contexts (Day et al., 2005). Conceptually, fatherhood is not a unitary role but a composite of partially distinct dimensions that cultures weight differently. These include a provider dimension oriented to material security, a caregiving and emotional dimension oriented to nurturance and responsiveness, a moral and socializing dimension concerned with values and guidance, a disciplinary dimension concerned with authority and boundary-setting, and a symbolic-cultural dimension that locates the father within a particular social and familial order (Pleck, 2010). These dimensions are neither equally salient across contexts nor necessarily harmonious within a single father, and both personal history and cultural expectation may reconfigure them. The present study does not treat all of these dimensions equally; rather, it foregrounds the emotional-relational and symbolic-cultural dimensions of fatherhood, because it is primarily within these domains that the legacy of childhood trauma and the constraints of masculinity norms become salient. In the Turkish context in particular, where fatherhood has historically been organized around provision, authority, and discipline rather than emotional expression (Kuscul and Adamsons, 2022), this conceptual emphasis allows the analysis to remain attentive to the tension between inherited role definitions and the more emotionally engaged forms of fatherhood that participants describe. Ecological perspectives emphasize the importance of men’s childhood experiences and the cultural environments in which they live when explaining paternal behavior (Cabrera et al., 2014; Petts et al., 2018). From this perspective, fathering practices are influenced by a wide range of contextual and cultural factors that can shape paternal behavior in both positive and negative ways. Moreover, ecological approaches highlight the importance of examining men’s own childhood experiences and psychological struggles in understanding their fathering practices (Cabrera et al., 2014). Exploring how men interpret their experiences of fatherhood, particularly regarding the presence or absence of father figures in their own childhoods, may therefore provide valuable insights into the development of paternal behavior (East et al., 2014). For this reason, examining the fatherhood experiences of men with childhood trauma within their sociocultural contexts is important for understanding both the mechanisms of intergenerational transmission and the potential for intervention (East et al., 2014, 2020). Nevertheless, the literature still provides limited insight into how men with childhood trauma interpret their experiences of fatherhood and how these experiences shape their fathering roles. Furthermore, much of the existing research has been conducted in Western societies, leaving the experience of fatherhood across different cultural contexts relatively underexplored. In the Turkish context, fatherhood has commonly been characterized by provision, authority, and discipline rather than by emotional expression. This characterization, however, warrants more than passing reference, since the empirical literature on Turkish fatherhood is both substantial and pointed. Studies consistently document that traditional masculinity norms remain powerful structuring forces in family life despite ongoing social modernization, positioning the father as an authority figure and economic provider while discouraging overt emotional expression (Boratav et al., 2014; Eslen-Ziya et al., 2021). Survey evidence is particularly telling. In a large sample of Turkish fathers, traditional masculinity beliefs, especially those emphasizing emotional restrictiveness, were directly associated with diminished nurturing and caregiving involvement. In contrast, more egalitarian and modernist beliefs predicted greater emotional closeness with children (Kuscul and Adamsons, 2022). At the same time, this picture is not static; a growing body of work points to an emergent, more emotionally engaged model of fatherhood coexisting in tension with inherited norms (Eslen-Ziya et al., 2021). It is precisely this tension between a culturally sanctioned model of restrained, authoritative fatherhood and the more involved father many men aspire to become that makes the Turkish context a theoretically productive site for examining how childhood trauma is negotiated in fathering. Despite these cultural dynamics, studies that explore the fatherhood experiences of men with childhood trauma within such cultural contexts remain scarce. Addressing this gap may therefore contribute important insights to the existing literature.

### The present study

The present study investigates the relationship between childhood trauma and fatherhood using a qualitative approach to explore how fathers interpret and make sense of their experiences. By examining this relationship within Türkiye’s cultural context, the study seeks to offer a perspective that extends beyond the predominantly Western focus of existing research. Interpretative phenomenological analysis (IPA) provides a particularly suitable methodological framework for this purpose, as it focuses on how individuals interpret and make meaning of significant life experiences (Smith et al., 2009). Although IPA does not require allegiance to a single grand theory, it benefits from an explicit interpretative orientation that guides the researcher’s engagement with participants’ accounts. The present study adopts the intergenerational transmission of trauma as its principal interpretative lens, understood not as a deterministic mechanism but as a relational process in which early caregiving experiences inform, rather than dictate, later fathering (East et al., 2014; van der Kolk, 2005). Two further perspectives are positioned as contextual layers within this lens rather than as competing frameworks. An ecological perspective situates fathering within nested familial, social, and cultural systems, directing attention to the conditions under which transmission is amplified or buffered. A masculinity-norms perspective identifies one such condition, particularly relevant to fathers: the culturally organized expectations that shape how emotion, authority, and care are enacted in the paternal role. Together, these orientations frame the analysis without pre-determining its content. They sensitize the interpretation to how the legacy of childhood trauma, cultural models of masculinity, and the relational demands of fathering converge in lived experience. At the same time, the specific meaning of that convergence remains an empirical question for the participants’ accounts to answer. Accordingly, IPA was employed to explore how men with histories of childhood trauma experience fatherhood and how these experiences are reflected in their parenting practices within a specific cultural context. In line with this aim, the present study addresses the following research questions:

1. How do Turkish men with childhood trauma experience fatherhood?
2. In what ways do traumatic childhood experiences influence their fatherhood experiences?
3. What themes emerge when examining the fatherhood experiences of men with childhood trauma?
4. What do Turkish men with childhood trauma perceive as necessary for demonstrating positive fathering behaviors?

## Materials and methods

### Research design

This study was designed as a qualitative investigation aimed at exploring the fatherhood experiences of men who had experienced childhood trauma. The study adopted an interpretative phenomenological analysis (IPA) approach, which focuses on understanding how individuals make sense of their lived experiences (Smith et al., 2009). IPA is grounded in phenomenological, hermeneutic, and idiographic traditions. This methodological perspective emphasizes exploring how individuals interpret and attach meaning to significant life events, making it particularly well-suited to examining complex psychosocial phenomena such as trauma, identity, and parenting.

The primary aim of IPA research is to provide a detailed examination of the subjective meaning worlds of individuals who have experienced a particular phenomenon. Accordingly, IPA studies prioritize depth of analysis rather than breadth of sampling. For this reason, such studies typically rely on relatively small and homogeneous samples that allow for an in-depth exploration of participants’ experiential accounts (Smith et al., 2009). Consistent with this approach, the present study focused on obtaining rich, detailed narratives from participants who shared a common experiential background.

### Participants

Participants were selected using purposive sampling, a strategy commonly employed in qualitative research to identify individuals with direct experience of the phenomenon under investigation (Johnson and Christensen, 2014). Recruitment announcements were distributed via social media, and 65 fathers voluntarily participated in an initial screening. During this stage, participants completed the Childhood Trauma Questionnaire developed by Bernstein et al. (1994) and adapted into Turkish by Şar et al. (2012). The questionnaire scores were used to identify individuals who had experienced relatively higher levels of childhood trauma and were therefore more closely aligned with the research focus. It is important to clarify the sense in which homogeneity is understood here. Within IPA, sample homogeneity is defined in relation to the research question rather than by demographic uniformity. What matters is that participants share the experiential phenomenon under investigation, such that the inquiry illuminates a defined experience rather than aggregating disparate ones (Smith et al., 2009). The participants in the present study were homogeneous in precisely this experiential sense. All were men who had experienced childhood trauma and were currently fathers, yet they differed in occupation, child age, and marital status. Rather than undermining the analysis, this demographic variation was treated as contextual texture. It allowed the shared phenomenon of fathering after childhood trauma to be examined as it manifests across a range of life circumstances, strengthening the credibility of patterns that recurred despite such differences. At the same time, this variation limits the claims that can be made, an issue revisited in the limitations section.

Of the 65 fathers who completed the initial screening, those scoring at or above the established CTQ cutoff (≥35) were identified as eligible. From this eligible pool, participants were invited for interviews until the analytic depth characteristic of IPA was achieved; recruitment stopped when additional cases no longer yielded substantially new experiential material. This process yielded a final sample of 17 fathers. Regarding marital status, 12 participants (70.6%) were married, and 5 (29.4%) were divorced. The occupational distribution of the participants included two physicians (11.8%), five engineers (29.4%), four teachers (23.5%), and six self-employed workers (35.3%). This distribution reflects a degree of occupational diversity within the sample. Regarding their children, eight participants (47.1%) had daughters and nine (52.9%) had sons. The ages of the participants’ children ranged from 4 to 22 years. This wide age range suggests that participants experienced fatherhood across multiple developmental stages, including early childhood, middle childhood, adolescence, and early adulthood. In qualitative research, presenting participants’ demographic characteristics in detail contributes to a contextualized understanding of the findings. Therefore, the demographic structure of the sample was reported in detail to illuminate the social and life contexts in which the participants’ experiences of fatherhood were situated.

During the participant selection process, specific inclusion and exclusion criteria were applied. Only Turkish men who had experienced childhood trauma and who had at least one child were included in the study. In addition, several exclusion criteria were implemented to ensure that interviews could be conducted within a psychologically safe framework and that participants’ narratives could be examined independently from ongoing clinical conditions. Accordingly, individuals who reported having a psychiatric diagnosis, using psychiatric medication, or currently receiving psychological or psychiatric treatment at the time of the interviews were excluded from the study. The primary rationale for applying these criteria was to prevent intense emotional processes that might arise during the interviews from being conflated with an ongoing clinical treatment context, thereby allowing the study to focus specifically on participants’ subjective experiences of fatherhood. This approach is consistent with ethical considerations commonly recommended in qualitative research that addresses traumatic life experiences. Ensuring that participants were not currently undergoing psychological treatment also helped ensure that interviews were conducted in a safe research context and enabled the independent interpretation of participants’ narratives from clinical intervention processes. This study was conducted in accordance with the ethical principles outlined in the Declaration of Helsinki. Ethical approval for this study was obtained from [redacted for anonymized review]. All participants provided informed consent before their involvement in the study and were informed of their right to withdraw at any time without consequence.

### Measures

#### Childhood trauma questionnaire

Childhood trauma was assessed using the Childhood Trauma Questionnaire (CTQ), originally developed by Bernstein et al. (1994) and later adapted into Turkish by Şar et al. (2012). The instrument consists of 28 items organized into five subscales assessing different forms of childhood maltreatment. Higher scores on the scale indicate greater levels of exposure to childhood traumatic experiences.

Within the scope of the present study, the internal consistency reliability coefficient of the scale was calculated as Cronbach’s alpha = 0.86, indicating satisfactory reliability. For the Turkish version of the CTQ, a cutoff score has been established to indicate the presence of clinically meaningful childhood trauma. According to this criterion, a total score of 35 or higher suggests a history of childhood trauma (Şar et al., 2012). In the current study, the scale was used as a screening tool to identify participants who had experienced relatively higher levels of childhood trauma and whose experiences were therefore relevant to the research focus. This use of a standardized instrument within an interpretative phenomenological design warrants brief justification, since IPA privileges participants’ own meaning-making over external measurement. The CTQ was not employed to define, quantify, or validate participants’ trauma, nor did its scores enter the analysis in any way. It functioned solely as a screening gate at the recruitment stage, a pragmatic means of identifying men for whom childhood maltreatment was likely to be a salient lived reality, thereby ensuring that interviews would engage the phenomenon of interest rather than relying on participants’ unaided self-categorization, which can be inconsistent for experiences as definitionally contested as childhood trauma. Once participants were included, the cutoff played no further role. The phenomenon under study was their own narrated experience of fatherhood, interpreted on its own terms. Understood this way, the instrument supports rather than supplants the phenomenological commitment, functioning as a recruitment filter analogous to the purposive selection criteria routinely applied in IPA studies.

#### Semi-structured interview questions

Data for the qualitative component of the study were collected using a semi-structured interview guide developed by the researchers. During the development of the interview protocol, the existing literature on childhood trauma, parenting experiences, fatherhood roles, and intergenerational transmission was first reviewed. Based on this review, an initial pool of interview questions was generated to align with the study’s research aims and questions.

The preliminary interview questions were subsequently evaluated by two academics with expertise in qualitative research methods and trauma studies. Based on their feedback, the scope and wording of the questions were revised and refined, resulting in the final version of the interview guide.

The interviews were conducted in a semi-structured format using open-ended questions, allowing participants to describe their experiences in their own words and in greater depth. During the interviews, follow-up questions were asked when necessary to clarify or further explore participants’ narratives. Such an approach is widely considered appropriate for interpretative phenomenological analysis (IPA) studies, as it enables researchers to gain deeper insight into how participants interpret and attach meaning to their lived experiences.

The semi-structured interview guide included the following core questions:

1. What does being a father mean to you? How would you describe your overall experience of fatherhood?
2. When you reflect on your childhood experiences, which memories stand out as the most influential or meaningful for you today?
3. How would you describe your relationship with your own father? In what ways do you think this relationship has influenced your understanding of fatherhood?
4. How do you experience your role as a father in everyday life? How would you describe yourself as a father in your relationship with your child?
5. Do you think your childhood experiences influence your current parenting behaviors? If so, in what kinds of situations do these influences become noticeable?
6. Are there moments in the fatherhood process when you feel challenged or emotionally triggered? How do you typically cope with these situations?
7. Are there particular things you pay attention to in order to provide your child with experiences different from those you had in your own childhood? In what ways have you tried to change or act differently in your role as a father?

During the interviews, additional probing questions such as “Could you elaborate on that?” “What did you feel at that moment?” and “What does this experience mean to you?” were used to deepen participants’ responses. These prompts helped participants reflect more extensively on their experiences of fatherhood, the connections they drew between their childhood histories and their parenting practices, and the meanings they attributed to these experiences.

### Data collection

Data were collected through individual semi-structured interviews conducted online via Zoom. The decision to conduct interviews online was based on two primary considerations. First, online interviews allowed greater flexibility in accommodating participants’ schedules. Second, discussing sensitive topics such as traumatic childhood experiences may be easier when participants can share their experiences in environments they perceive as safe and private. For these reasons, online interviewing was considered an appropriate data collection strategy, particularly in qualitative studies addressing emotionally sensitive experiences.

Each interview lasted between 45 and 75 min. This duration provided sufficient time for participants to reflect on and describe their experiences in depth, while also helping to prevent excessive emotional burden during the interview process. The semi-structured interview format allowed the researcher to focus on key research topics while also providing participants with the flexibility to narrate their experiences within their own interpretative frameworks. Such flexibility is consistent with the interpretative phenomenological analysis (IPA) approach, which seeks to explore individuals’ subjective experiences and the meanings they ascribe to them.

During the interviews, participants were invited to reflect on their childhood experiences; the perceived influence of traumatic experiences on their current fathering practices; their relationships with their children; moments of emotional triggering; the balance between authority and emotional closeness in fatherhood; and experiences of change or transformation in their fathering roles. Throughout the interviews, the interviewer aimed to avoid interrupting participants’ narratives, use non-directive language, and remain attentive to each participant’s emotional pace. When appropriate, follow-up questions were asked to explore participants’ accounts further and clarify the contextual, emotional, and interpretative dimensions of their experiences.

### Data analysis

The data were analyzed using interpretative phenomenological analysis (IPA) following the methodological guidelines outlined by Smith et al. (2009). The analytic process was conducted through several iterative stages. In the first stage, all interview recordings were transcribed verbatim. The transcripts were then read repeatedly by the primary researcher to develop a deep familiarity with the data. During this stage, particular attention was given to significant statements, emotional expressions, and recurring patterns of meaning within participants’ narratives. In the second stage, detailed initial notes were generated for each transcript. These notes included descriptive, linguistic, and conceptual observations. Based on these preliminary annotations, emergent themes were developed that captured key aspects of participants’ experiences. This stage involved both descriptive interpretations of participants’ accounts and more conceptual reflections aimed at identifying underlying meanings. In the third stage, the themes identified for each participant were examined in relation to one another to construct hierarchical thematic structures comprising superordinate and subordinate themes. Following this within-case analysis, cross-case comparisons were conducted to identify shared thematic patterns across participants’ narratives. Throughout the analytic process, the qualitative data analysis software MAXQDA was used to organize, code, and manage the data.

### Trustworthiness, reliability, and credibility

Several strategies were implemented to enhance the credibility and rigor of the study. First, the research questions were reviewed not only by the primary researcher but also by two independent researchers. This review process helped evaluate whether the questions were conceptually aligned with the research phenomenon, clearly articulated, and free from leading assumptions. As a result, the study’s conceptual framework underwent collective academic scrutiny prior to data collection. Second, the coding and theme development process was not based solely on a single researcher’s interpretations. In addition to the primary researcher, two additional researchers independently participated in the coding process. The resulting codes and themes were subsequently compared and discussed. When discrepancies or ambiguities emerged, the researchers engaged in collaborative discussions until a shared interpretative understanding was reached. Rather than seeking a purely mechanical percentage agreement, this process aimed to achieve interpretative consensus consistent with the epistemological foundations of qualitative research. In IPA studies in particular, such collaborative analytic discussions are considered an important strategy for enhancing analytical depth.

Third, the findings were supported by direct quotations from participants. Including participants’ own words allows readers to observe how themes were derived from the data and provides transparency between the raw data and the researchers’ interpretations. Such transparency is widely regarded as a key component of qualitative credibility. To strengthen the dependability of the research process, both data collection and data analysis procedures were implemented systematically. Interviews were conducted using a semi-structured framework that consistently addressed core research areas across participants while allowing flexibility to explore participants’ narratives in greater depth. The analytic process followed a clearly structured sequence, including transcription, repeated reading of transcripts, initial note-taking, identification of emergent themes, within-case analysis, and cross-case thematic comparison. The involvement of multiple researchers in the coding and theme development process also contributed to the methodological stability of the analysis. Intercoder agreement was assessed using the formula proposed by Miles and Huberman (1994): Reliability = Agreement/(Agreement + Disagreement). Based on this calculation, the intercoder agreement index was 0.84. In addition, the themes were supported by direct quotations from participants to ensure that the analytic interpretations remained closely grounded in the data. To demonstrate that the findings did not merely reflect the researchers’ prior assumptions, interpretations were continuously compared with the empirical data throughout the analysis. When developing themes, explicit evidence from the interview transcripts was sought for each interpretation, and overly broad or unsupported conclusions were avoided. The involvement of multiple researchers in the coding process further reduced the influence of individual researcher bias and strengthened confirmability. Disagreements were resolved through discussion, allowing themes to be refined while remaining closely tied to the data.

Reflexivity was also considered an important component of the analytic process. Topics such as trauma and fatherhood carry significant emotional and cultural meaning, making it essential for researchers to remain aware of their own assumptions and interpretative positions. Throughout the analysis, the research team engaged in regular analytic discussions to reflect on their theoretical perspectives and potential biases. By continuously comparing interpretations with the data, the researchers sought to ensure that participants’ narratives were interpreted in ways that remained grounded in the participants’ own accounts rather than the researchers’ preconceptions.

The aim of the present study was not to produce statistically generalizable findings. Instead, the study sought to generate a context-sensitive and in-depth understanding of the fatherhood experiences of men who had experienced childhood trauma. To support transferability, detailed descriptions were provided of participant selection, the data collection context, the interview process, and theme development. This level of contextual detail allows readers to evaluate the conditions under which the findings were generated and to assess the extent to which the results may apply to other contexts.

## Results

The fatherhood experiences of Turkish men with a history of childhood trauma were examined using an interpretative phenomenological analysis (IPA) approach. The analysis revealed six overarching themes that shaped participants’ experiences of fatherhood. These themes suggest that emotional wounds originating in childhood often become reactivated within the fathering role. At the same time, culturally embedded norms of masculinity appear to constrain the expression of emotions in the context of fatherhood. Nevertheless, the findings also indicate that for some fathers, the experience of parenting may create opportunities for reflection, personal change, and transformation. Together, the themes illustrate how fatherhood can function both as a space where unresolved childhood emotional experiences resurface and as a context in which individuals begin to reinterpret and renegotiate them. It should be noted that although the themes are presented in a sequence that moves from the reactivation of early wounds toward processes of change, this ordering is analytic rather than chronological. It does not imply that participants progressed through fixed stages; the themes coexisted, overlapped, and recurred within individual accounts rather than unfolding as a linear developmental sequence. A summary of the themes and their corresponding subthemes is presented in Table 1. Each theme and its corresponding subthemes are presented below and explained in detail with illustrative quotations drawn from participants’ narratives.

**Table 1:** Themes, subthemes, and illustrative quotations.

Theme	Subthemes	Illustrative quotation
Early emotional wounds: the emotional legacy of the past	Emotional neglect, insecure home environment, conditional love, difficulty establishing emotional closeness, learned fatherhood patterns, shame and self-blame	*“It is like there is an automatic ‘father mode’ inside me, and it is not easy to break out of it.”*
The silent rules of masculinity	Suppressing emotional expression, the obligation to appear strong, silent communication	*“Even when I feel upset, I do not show it… the same reaction still comes automatically.”*
Authority and emotional closeness in fatherhood	The weight of the father role, between discipline and affection, maintaining emotional distance, becoming a more emotionally present father	*“When I get too close, it almost feels like my authority might disappear.”*
Moments of emotional triggering	Triggering through daily stress, reduced tolerance, recognizing similarities with one’s own father, triggered by children’s behavior, regret afterward	*“I realized I was saying exactly what my father used to say. That moment felt really disturbing.”*
The beginning of transformation in fatherhood	Trying to regulate oneself, connecting present reactions to the past, trying to understand the child, trying new ways of responding	*“Now, when that moment comes, I try to pause… once I calm down a bit, I talk.”*
Changing experiences of fatherhood	Learning to establish emotional closeness, managing reactions, redefining fatherhood, the desire to interrupt intergenerational transmission	*“I want what came to me from my father to end here.”*

### Theme 1: early emotional wounds: the emotional legacy of the past

This theme reflects how participants’ experiences of fatherhood were deeply shaped by emotional wounds originating in their own childhoods. Rather than understanding fatherhood solely through their current parental responsibilities, participants frequently interpreted their fathering experiences through the emotional traces of their early life experiences. In their narratives, experiences such as emotional neglect, an insecure family environment, and conditional expressions of love emerged as a persistent “emotional legacy” that continued to influence their approach to fatherhood. For some fathers, this emotional legacy created a sense of vulnerability, making it difficult to establish emotional closeness. For others, these experiences were associated with enduring internal burdens, including feelings of shame and self-blame. In this sense, fatherhood was experienced as a life context in which participants re-encountered their past, where early emotional wounds could be both reactivated and, in some cases, gradually reinterpreted and transformed.

*Emotional Neglect* “If someone had come to me as a child and simply asked, ‘Are you okay?’, maybe things would have been different. However, no one ever did. Now I look into my son’s eyes and want him to know that he is understood.”

*Insecure Home Environment* “There was never any real peace in our house. You never knew what kind of mood my father would come home in. I grew up constantly on alert. Sometimes I am afraid of feeling that same tension in my own home now.”

*Conditional Love* “To be loved, I had to behave well. When I was successful, I was valued; when I made a mistake, I became invisible. As a father, I pay particular attention to this. I do not want my child to feel that love has to be earned.”

*Difficulty Establishing Emotional Closeness* “I want to hug my child, but sometimes my hand does not move. Something inside tells me not to get too close. Later, I realized that when I was a child, there was no one I could lean on, either.”

*Learned Fatherhood Patterns* “Sometimes I am surprised by the words that come out of my own mouth. I hear myself repeating the same things my father used to say. It is like there is an automatic ‘father mode’ inside me, and it is not easy to break out of it.”

*Shame and Self-Blame* “For a long time, I thought the problem was me. I kept asking myself, ‘Why am I like this?’ When I became a father, that old sense of shame sometimes came back. However, now I understand that it is not my fault; it is the weight of the past.”

### Theme 2: the silent rules of masculinity

This theme reflects how participants’ experiences of fatherhood were shaped by internalized, often unspoken norms of masculinity. Many fathers described growing up in family environments where emotional expression was limited or discouraged. Over time, suppressing emotional expression became a habitual response that continued into their experiences of fatherhood. For many participants, masculinity was closely associated with the expectation of remaining strong and emotionally controlled. Expressions of vulnerability such as sadness, emotional need, or distress were often perceived as incompatible with traditional masculine roles. As a result, participants frequently described difficulties in openly expressing affection or emotional closeness toward their children, even when they deeply cared about them. Instead of expressing love verbally or through overt emotional communication, many fathers reported relying on more indirect or silent forms of connection with their children. In this sense, fatherhood was often experienced as an emotional presence conveyed through actions rather than through explicit expressions of feeling.

*Suppressing Emotional Expression* “Even when I feel upset, I do not show it. It was like that when I was a child, and now that I am a father, the same reaction still comes automatically.”

*The Obligation to Appear Strong* “Being a father means you have to stay strong. Even if I am exhausted, I try not to show it to my child.”

*Silent Communication* “I love my child, but I cannot always say it out loud. Most of the time, I stay quiet and sit beside him.”

Taken together, these narratives suggest that for many participants, fatherhood was not experienced solely as a parenting role but also as an emotional position shaped by internalized cultural expectations about masculinity. Whereas the present theme concerns masculinity as an internalized property of the self, a habituated disposition toward emotional restraint carried forward from the participant’s own upbringing. By contrast, the subsequent theme addresses a distinct analytic object, namely the structural dilemma of the paternal role itself, in which authority and closeness are experienced as competing demands. These silent norms often structured how fathers expressed care, closeness, and responsibility within their relationships with their children.

### Theme 3: authority and emotional closeness in fatherhood

Participants frequently described fatherhood not merely as a parenting responsibility but also as a socially defined role that carries expectations, responsibilities, and a certain symbolic weight within the family. This theme is therefore not a restatement of internalized masculine restraint but a shift in analytic focus from disposition to position. Here, the tension is located in the father role as a relational structure, where the perceived requirement to preserve authority is set against the wish for emotional proximity, producing a dilemma that participants actively negotiate rather than passively enact as a habit. Within the cultural context in which many participants were raised, being a father was associated with maintaining authority, responsibility, and a stable presence within the household. In participants’ narratives, the perceived weight of the father role was often intertwined with the challenge of balancing discipline and affection. Several fathers expressed a desire to build emotionally closer relationships with their children yet reported being influenced by traditional expectations that fathers should maintain a certain emotional distance to preserve authority. However, this theme also suggests that fatherhood was not experienced as a fixed or unchanging role. Alongside inherited patterns shaped by authority and distance, many participants expressed a growing desire to redefine their role as fathers. In particular, some fathers described an effort to become more emotionally accessible and relationally present with their children, indicating an ongoing negotiation between traditional paternal norms and emerging forms of fatherhood.

*The Weight of the Father Role* “Being a father feels like more than just raising a child. You carry a certain presence in the house. Everyone expects something from you. Sometimes that weight really sits on your shoulders.”

*Between Discipline and Affection* “I love my child very much, but sometimes I feel like I have to be strict. I want to raise my child with love, but the idea of discipline is always there in the back of my mind.”

*Maintaining Emotional Distance* “My father was always distant. I want to be different, but sometimes I find myself standing in the same place. When I get too close, it almost feels like my authority might disappear.”

*Becoming a More Emotionally Present Father* “I do not want my child to feel afraid of me. There was always a wall between my father and me. I am trying to learn how to be a father without building that wall.”

### Theme 4: moments of emotional triggering

Participants frequently described situations in everyday family life in which their emotional responses were rapidly triggered. Daily stress, fatigue, and minor tensions within the household were often reported to lower emotional tolerance and increase the likelihood of reactive responses. During such moments, fathers reported reduced patience, sudden emotional reactions, and difficulty maintaining self-control. Many participants reported that a striking realization often followed these moments. They recognized themselves behaving in ways that resembled their own fathers. For several fathers, this realization was emotionally unsettling and prompted reflection on the possibility of repeating patterns from their own childhoods. Participants also identified specific child behaviors, such as crying, testing boundaries, or demanding attention, as situations that could intensify emotional triggering. These moments were frequently described as emotionally complex, as feelings of regret often followed immediate reactions. Fathers described these experiences as critical moments when the possibility of intergenerational transmission became apparent, highlighting the tension between inherited patterns and the desire to respond differently.

*Triggering Through Daily Stress* “When I come home from work exhausted, I get triggered more easily. Things that normally would not bother me suddenly feel much bigger.”

“When stress builds up during the day, I become more sensitive at home. My child can sense it as well.”

*Reduced Tolerance* “Sometimes my patience suddenly drops. Afterward, I am surprised at myself and wonder why I reacted so strongly. It is usually something very small, but in that moment, I feel like I have run out of patience. Later, I regret it.”

*Recognizing Similarities with One’s Own Father* “I said something, and then I stopped… I realized I was saying exactly what my father used to say. That moment felt really disturbing.”

“When my tone of voice changes, I notice it. I start wondering, ‘Am I becoming like my father?’”

*Triggered by Children’s Behavior* “When my child starts crying, something tightens inside me. In those moments, it becomes harder to stay calm.”

“When my child pushes boundaries, I get triggered. As a child, I was never allowed to do that.”

*Feelings of Regret Afterward “The moment passes, but afterward* I sit with the feeling and feel sad. I wish I had reacted differently.”

“When I look at my child afterward, I feel regret. I do not want the same cycle to repeat.”

### Theme 5: the beginning of transformation in fatherhood

As described in the previous themes, many fathers reported that the effects of their childhood trauma became particularly visible during emotionally triggering moments. However, for some participants, this awareness did not remain merely a difficult confrontation with the past. Instead, it marked the beginning of a process in which they reconsidered and gradually reshaped their responses as fathers. Participants described becoming increasingly aware of the automatic reactions they had internalized during their childhood. When these reactions surfaced, some fathers reported consciously attempting to regulate their responses. They described reflecting on possible links between their past experiences and their current parenting behaviors, while also working to understand their children’s emotional needs better. Importantly, this process was rarely described as a rapid or effortless change. Rather, participants described it as a gradual, sometimes challenging process of learning and self-reflection that required conscious effort and ongoing awareness.

*Trying to Regulate Oneself* “In the past I would react immediately. Now, when that moment comes, I try to pause. Sometimes I even count to myself, and once I calm down a bit, I talk.”

“When my child does something now, I try to hold myself back first. I take a breath before responding.”

*Connecting Present Reactions to the Past* “Sometimes I ask myself why I became so angry in that moment. Then I start thinking that maybe it has something to do with my childhood.”

“When I notice how I react to my child, I start asking myself where I learned this. Most of it seems to go back to my childhood.”

*Trying to Understand the Child* “In the past, when my child cried, I would immediately try to stop it. Now I try to understand why he is crying first.”

“Sometimes I remind myself that he is also a small human being. He has moments when things are difficult for him, too.”

*Trying New Ways of Responding* “Sometimes I intentionally try to act differently so that I will not become like my father.”

“In the past, I would react harshly right away. Now I try to solve things by talking.”

### Theme 6: changing experiences of fatherhood

Participants acknowledged that the influence of childhood trauma and internalized norms of masculinity could not be easily eliminated. Nevertheless, many fathers described gradually developing new perspectives and attitudes toward fatherhood as their parenting experiences unfolded. For several participants, becoming a father created an opportunity to reconsider previously learned relational patterns and to respond differently in their relationships with their children. A recurring idea in participants’ narratives was the desire to prevent the transmission of their own traumatic experiences to the next generation. Fathers frequently expressed a strong motivation to ensure that their children would not grow up under the same emotional conditions they had experienced in their own childhoods. In this sense, fatherhood was not only experienced as a space where past experiences resurfaced, but also as a context in which change and reinterpretation became possible. The narratives suggest that fatherhood can serve as a developmental turning point, prompting participants to renegotiate their emotional practices and gradually move toward more emotionally engaged and reflective forms of parenting.

*Learning to Establish Emotional Closeness* “In the past, keeping distance felt normal. Now I am learning to be closer to my child.”

“Hugging, talking… those things used to feel unfamiliar to me, but they are changing with fatherhood.”

*Managing Reactions* “There are still moments when I struggle, but now I try to hold myself back.”

“Before, I would react immediately. Now I try to pause and calm down first.”

*Redefining Fatherhood*: “I am starting to realize that being a father is not only about being strict.”

“Sometimes being there for your child means understanding, not discipline.”

*The Desire to Interrupt Intergenerational Transmission* “I do not want what I experienced to pass on to my child. That is my biggest motivation.”

“I want what came to me from my father to end here.”

The vast majority of participants described fatherhood as a process in which childhood traumas resurface and require confronting past experiences. However, while a small number of participants acknowledged experiencing neglect, physical abuse, or emotional trauma in childhood, they explicitly distanced themselves from the idea that these experiences determined their current fathering behavior. What was important for these participants was to focus on present parenting choices and to treat their own children differently, rather than constantly re-evaluating the past. One participant described the situation with the following words:

*“Yes, I have childhood trauma; my father always neglected me, but I am not sure that affected my whole life. I am a father myself now. I am not interested in what my father does anymore.”*

Similarly, another participant described the absence of their father during childhood as a negative experience but stated that they did not consider it a defining part of their life today.

*“My father worked very hard. I saw him so little. Nevertheless, it did not affect me badly. I just learned to enjoy the time he was home and not expect too much from him.”*

Some participants, however, viewed past experiences of physical violence separately from their current fatherly behavior. For example, one participant stated:

*“I was subjected to violence by my father. I cried then, and it passed. He was an old-fashioned man; I do not expect him to know anything about parenting. His violence towards me did not affect my fathering. I do not hit my children.”*

Similarly, another participant emphasized that their childhood experiences did not determine their current parenting style:

*“My father was not a good man. He left very traumatic marks on my brother and me. However, I do not think that has affected my fathering. I mean, it does not even cross my mind most of the time. The past is in the past.”*

Another participant, without denying the traumatic experiences he had lived through, stated that he did not want them to be central to his life.

*“My father used to swear at me a lot and even beat me occasionally. He was a tough man. However, I do not talk about it much because of my childhood trauma. Because it depends on how you want to perceive it. I am already working very hard. I do not want to upset myself by dealing with such things. I try my best to treat my child properly.”*

These narratives do not indicate that childhood trauma was ignored, but rather that the participants’ relationship with their past experiences has changed. While participants in the dominant pattern described fatherhood as an experience in which childhood wounds resurfaced, participants in this group consciously tried to separate the past from the present; they framed this as a personal choice not to allow their childhood experiences to define their current fatherly identities. Therefore, the same life event was interpreted differently by the participants: for some fathers, the past persisted as a constantly relived experience, while for others, it was framed as a period of life left behind and separated from their current parenting preferences.

## Discussion

The present study explored how Turkish men with childhood trauma histories experience fatherhood. The findings reveal that fatherhood functions as a relational context in which childhood wounds are reactivated, shaped by cultural norms, and for some fathers, gradually renegotiated (Widom et al., 2015).

### Intergenerational transmission and the role of shame

Participants did not passively replicate their fathers’ behaviors but experienced intergenerational patterns with surprise and internal conflict, suggesting transmission operates through implicit schemas that become visible only within caregiving, consistent with longitudinal evidence that aggressive and harsh relational patterns experienced in the family of origin predict similar patterns in adult relationships, with transmission partially mediated by what was directly experienced as a child rather than consciously modeled (Cui et al., 2010; Brown et al., 2018). This conflict may represent a first disruption in the cycle’s automaticity. Closely bound to this conflict was pervasive shame, which emerged in our findings as a paradoxical force that simultaneously fueled fathers’ desire to parent differently and obstructed the very vulnerability that emotional closeness requires. This dual function is consistent with long-standing theoretical distinctions in the shame literature, where shame, unlike guilt, is understood to target the self rather than the behavior, producing withdrawal, concealment, and defensive distancing rather than reparative action (Tangney and Dearing, 2002). In the context of childhood adversity, shame has been described as one of the most persistent and disabling affective residues, and recent meta-analytic evidence confirms robust associations between child maltreatment and later shame, with the strongest effects observed for emotional abuse and neglect (Zhang et al., 2025).

The present study’s focus on shame as a paradoxical force resonates with this broader picture. However, it highlights a specifically paternal dimension of shame that motivates fathers to break the cycle yet may simultaneously constrain the emotional availability needed to sustain that break. This is particularly consequential given developmental evidence that paternal emotional availability and responsiveness are associated with adolescent emotion regulation and resilience (Özaydın and Soyyiğit, 2024); shame-driven withdrawal, even when motivated by protective intent, may inadvertently reproduce the emotional distance fathers themselves experienced as children. Notably, while studies of mothers with childhood histories of abuse and neglect have documented comparable mechanisms where unresolved trauma and limitations in trauma-specific mentalization predict intergenerational continuity of insecure attachment (Berthelot et al., 2015), the expression of these dynamics in fathers is likely further shaped by gendered norms that prohibit the very acknowledgment of vulnerable affect, a point we return to below.

### Habitus and the doubled constraint of trauma and masculinity

Drawing on Bourdieu’s concept of family habitus, the automatic reproduction of parenting patterns observed in the present study can be understood as dispositional regularities that operate below the level of conscious awareness, producing history based on history [Bourdieu, 1990, quoted in Brannen (2015)]. Conceived this way, the trigger–reaction moment described by participants can be read as the habitus in operation. The father’s inherited dispositions, laid down in his own childhood, are enacted automatically and pre-reflectively before deliberation intervenes. What participants experienced as an “automatic father mode” corresponds closely to this notion of embodied, durable dispositions that reproduce the past in the present without conscious intent. Crucially, however, the recognition–regret component suggests that habitus is not wholly deterministic. The moments in which participants caught themselves and felt the dissonance of acting ‘like their own fathers’ represent precisely those junctures at which dispositions are rendered visible and therefore become, at least potentially, open to revision. These vulnerabilities were compounded by culturally internalized masculinity norms, producing a synergistic interaction in which trauma and culture amplified one another. As established in the introduction, traditional masculinity norms in Turkey position the father as an authority figure and provider while discouraging emotional expression, a pattern quantitatively confirmed among Turkish fathers (Kuscul and Adamsons, 2022). This pattern is consistent with the broader hegemonic masculinity framework (Connell and Messerschmidt, 2005), in which dominant forms of manhood require the active suppression of vulnerability, emotional expression, and physical tenderness, precisely the capacities that responsive caregiving demands (Magaraggia, 2013). Qualitative evidence from other cultural contexts converges on this point: Cleary’s (2022) work on emotional constraint demonstrated that men raised in hegemonic masculine environments frequently struggled to engage emotionally with their own sons, reproducing, across generations, the very emotional distance they had experienced as children. What makes the Turkish case distinctive, however, is not merely the presence of such norms but their intersection with childhood trauma. The combination of a cultural prohibition on vulnerable affect with an unprocessed history of childhood adversity may produce a doubled constraint on emotional availability: trauma generates the affective material that would need to be processed, while masculinity norms restrict the very channels of disclosure, tenderness, and help-seeking through which such processing typically occurs. This intersection represents a dimension insufficiently addressed in Western-focused intergenerational transmission literature, which often treats masculinity and trauma as separable variables rather than mutually reinforcing forces.

### The trigger–reaction–recognition–regret sequence

**Figure 1:**
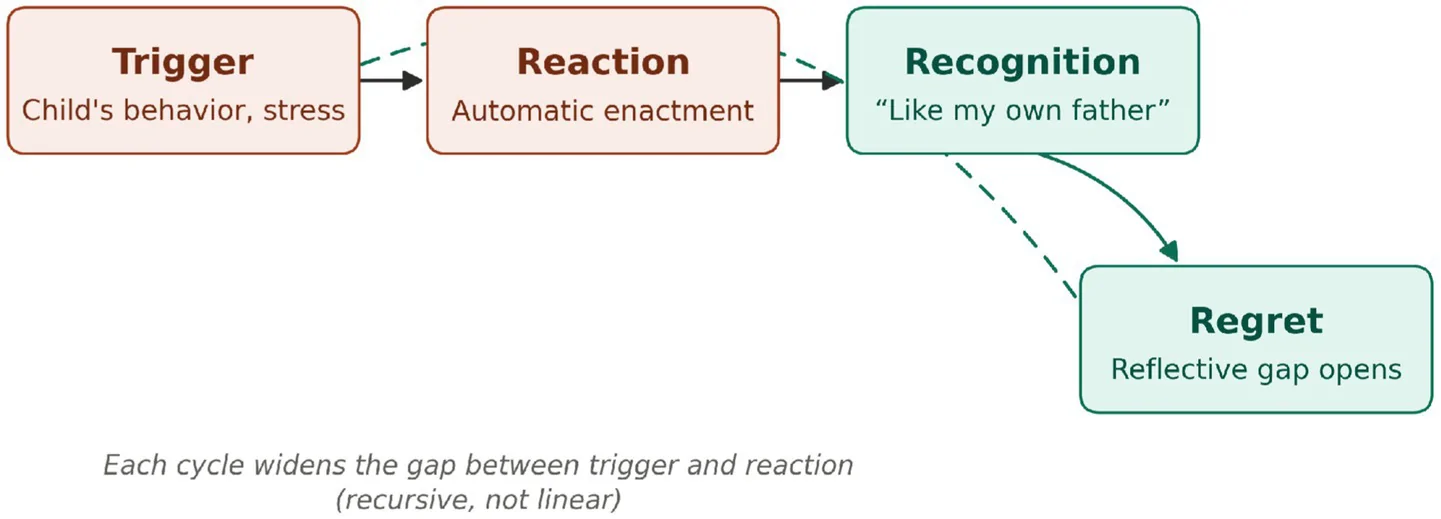
The trigger–reaction–recognition–regret sequence. The coral stages mark the automatic phase, in which a trigger provokes a pre-reflective reaction; the teal stages mark the reflective phase, in which the father recognizes the inherited pattern and experiences regret. The dashed return path indicates the recursive, non-linear nature of the process: rather than a single staged progression, the sequence may repeat, with each cycle potentially widening the gap between trigger and reaction.

The most distinctive finding of the present study was the trigger–reaction–recognition–regret sequence (Figure 1), a micro-level temporal mechanism through which intergenerational transmission both occurs and, paradoxically, becomes interruptible within the same relational episode.

The trigger–reaction component of this sequence may be interpreted, within a mentalization framework, as a momentary collapse of parental reflective functioning, wherein the father’s own unprocessed trauma temporarily forecloses access to his child’s mental states, substituting reactive enactment for mentalized response (Slade, 2005). It should be emphasized that reflective functioning was not directly assessed in the present study; this framing is offered as a theoretical interpretation of participants’ accounts rather than as a measured finding. This reading is consistent with evidence that reflective functioning is not globally stable but domain-specific, often remaining intact under ordinary circumstances while collapsing precisely in contexts that resonate with unresolved trauma (Berthelot et al., 2015). Understood this way, what participants described in the trigger–reaction moment appears less like a deficit of parenting skill or knowledge than a temporary loss of the capacity to hold the child’s mind in mind. This capacity may be particularly vulnerable when the child’s behavior activates implicit schemas rooted in the father’s own history of adversity. The resulting reaction feels, to the father, automatic and alien at once. It is recognizably his, yet recognizably not the parent he intends to be. This phenomenological duality is what makes the sequence diagnostic of intergenerational transmission rather than ordinary parental frustration; it reveals the presence of a second, historical relationship superimposed on the present one.

The recognition–regret component of the sequence may be the more generative in theoretical terms. It can be read as the moment at which automaticity is ruptured, and reflective capacity is, however briefly, restored. That this awareness tended to emerge within dysregulation rather than after calm reflection is paradoxical but consistent with mindful parenting models, which conceptualize awareness not as a precondition for parenting well but as a capacity cultivated through repeated contact with precisely the moments in which one has parented badly (Duncan et al., 2009). Within this framework, the regret that follows a reaction is not merely an affective consequence but a cognitive-affective signal that interrupts what Dumas (2005) termed the automaticity of maladaptive parenting interactions, thereby creating a small temporal gap in which alternative responses become possible. Our findings suggest that for some fathers, this gap gradually widened, with the motivation to prevent transmission serving as the strongest engine of change, echoing Shafer and Easton’s (2021) finding that adverse childhood experiences, when integrated rather than avoided, can mobilize rather than merely impair fathering behaviors. However, change was neither universal nor reliably sustained without external scaffolding. This pattern aligns with longitudinal and meta-analytic evidence that the intergenerational cycle of maltreatment is most reliably interrupted in the presence of safe, stable, nurturing relationships, whether with a romantic partner, a therapist, or another supportive adult, rather than through individual insight alone (Jaffee et al., 2013; Schofield et al., 2013). In other words, fatherhood appears to create the conditions under which intergenerational patterns can become visible and questionable, but translating that visibility into durable change typically requires relational and therapeutic supports that most fathers in our sample lacked. This is consistent with Brannen’s (2015) observation that while intergenerational transmission implies continuity, each generation places its own mark upon what is passed on, producing both repetition and transformation, a dynamic captured by the partial and fragile yet genuinely meaningful changes our participants described. However, the participant narratives did not form a wholly uniform pattern. A minority of fathers, while openly acknowledging childhood neglect, physical abuse, or emotional trauma, resisted the idea that these experiences determined their present fathering, framing the deliberate separation of past from present as a conscious parenting stance rather than evidence of unresolved wounds. Read through an interpretative phenomenological lens, this divergence is not a contradiction of the dominant pattern but a reminder that the same early experience can be inhabited in markedly different ways. It suggests that the relationship between childhood trauma and fatherhood is neither linear nor inevitable and that convergence on a triggered, conflicted mode of fathering, however prevalent in this sample, should not be mistaken for the only possible trajectory.

### Toward a conceptual framework

When the threads of this study are woven together, a conceptual framework for understanding fatherhood among Turkish men with a history of childhood trauma emerges. This framework is shaped around three interrelated forces: the emotional legacy of early-life adversity, culturally internalized norms of masculinity, and the relational demands of fatherhood. Although fatherhood in Turkey has evolved with modernization, it is still shaped within traditional, hierarchical, and gendered family structures; thus, the emotional legacy of childhood trauma is not merely an individual psychological burden but also an experience that takes on new meaning within culturally regulated expectations of masculinity and fatherhood (Çorapçı et al., 2025; Kisbu-Sakarya et al., 2022; Sen et al., 2014).

These three forces intersect at points we define as “triggering turning points.” During daily interactions, the child’s behavior triggers implicit schemas rooted in the father’s past; meanwhile, the fact that constructs of masculinity in Turkey are often defined through toughness, self-sufficiency, and emotional control simultaneously limits the processing of such activation through relational resources (Cleary, 2022; Tekkas Kerman and Betrus, 2019). The cultural tension between the desire to form a close bond with the child and the expectations of hegemonic masculinity transforms these turning points into thresholds where not only is traumatic memory activated, but pressures regarding how masculinity should be performed also intensify (Eslen-Ziya et al., 2021; Petts et al., 2018).

At these turning points, two distinct paths emerge. The first path—reenactment—is characterized by the father adopting a collapsed mindset and reproducing patterns he did not consciously choose. The fact that patriarchal masculinity norms in Turkey increase the risk of physical punishment (Kisbu-Sakarya et al., 2022) and that adherence to traditional masculinity norms is associated with less care and harsher discipline (Petts et al., 2018; Shafer et al., 2021) makes this path culturally more visible. Furthermore, emotional restraint in men—which limits the expression of distress and the seeking of help—further reinforces the cycle of reenactment (Cleary, 2022).

The second pathway, “emergent awareness,” begins with the underlying pattern of the current emotional dysregulation becoming visible; for some fathers, this visibility allows them to question and transform patterns inherited from the past. Findings indicating that many fathers in Turkey are consciously striving to break away from the legacy of distance and authority inherited from their own fathers (Çorapçı et al., 2025; Kuscul and Adamsons, 2022) lend cultural credibility to this second pathway. Indeed, national sample studies have shown that Turkish fatherhood profiles are not merely “distant and authoritarian”; a significant portion exhibits warmer and more authoritative patterns, and that spousal support and marital satisfaction are associated with these patterns (Çelik and Bulut, 2019; Kisbu-Sakarya et al., 2022). These findings suggest that the emerging path of awareness represents a feasible arena for transformation in Turkey—both psychologically and culturally.

When Turkey’s cultural context is added to this framework, fatherhood ceases to be merely a space where trauma is reproduced; it emerges as a contradictory yet transformable relational space shaped by patriarchal continuity, social change, class differences, and the search for emotional intimacy. While the traditional father figure perpetuates a hierarchical relationship that fosters respect and fear (Çorapçı et al., 2025; Kisbu-Sakarya et al., 2022), forms of fatherhood that are closer, more communicative, and emotionally accessible are gaining strength, particularly among urban and educated groups (Ghaffari and Ruspini, 2023; Kuscul and Adamsons, 2022). Class, education, spousal support, and the quality of intra-family relationships significantly shape how fatherhood is experienced; while an emphasis on authority and moral guidance predominates in low-SES contexts, support for autonomy and open expressions of love take center stage in higher-SES groups (Çorapçı et al., 2025). It has been demonstrated that gendered attitudes influence fathering participation through self-efficacy and relationship quality, while mothers’ education and employment are associated with fathers’ caregiving participation via their egalitarian beliefs (Enoz and Araz, 2024; Kuscul and Adamsons, 2022).

Consequently, Turkish men’s experience of fatherhood extends beyond being a space where trauma is reproduced; it emerges as a dynamic relational space where the legacy of authority and distance inherited from the past is negotiated alongside increasing expectations of emotional closeness and care. Therefore, the discussion can particularly emphasize that fatherhood in Turkey is a transitional space where both intergenerational repetition and the search for transformation occur simultaneously (Kisbu-Sakarya et al., 2022; Ghaffari and Ruspini, 2023; Yehuda and Lehrner, 2018).

### Clinical implications

The trigger–reaction–recognition–regret sequence provides a naturalistic template for mindfulness-based interventions, helping fathers extend the space between trigger and response. Shame and masculinity norms necessitate culturally sensitive designs framing participation as responsible fathering. The strong motivation to prevent transmission offers a powerful engagement lever. Given that the transformation remained fragile without professional support, structured programs may be critical for scaffolding a process that had begun organically. Emerging evidence demonstrates that trauma-informed parenting programs yield significant improvements in both parenting outcomes and parental mental health, supporting the clinical utility of structured interventions for trauma-affected parenting (Joseph-McCatty et al., 2025). Randomized controlled trial evidence further indicates that mindfulness-based interventions are promising in reducing parenting stress, with most programs derived from the MBCT and MBSR frameworks (Caetano et al., 2024; Zhang et al., 2021).

## Limitations

Several limitations of the present study warrant consideration. First, as is characteristic of IPA research, the purposive sample prioritizes analytic depth over statistical representativeness, and the findings should not be interpreted as generalizable to all Turkish fathers with childhood trauma histories. Second, the data rely on retrospective self-report, which is inherently subject to memory reconstruction and social desirability biases; participants’ accounts of their childhood experiences and current parenting behaviors may not fully reflect objective events. Third, the relatively educated occupational profile of the sample which included physicians, engineers, and teachers may limit the transferability of the findings to fathers from lower socioeconomic backgrounds or with less formal education. Fourth, the exclusion of fathers currently receiving psychological or psychiatric treatment, while ethically justified, may have resulted in a sample that underrepresents fathers whose trauma histories are more severe or whose parenting difficulties are more pronounced. Fifth, the cultural specificity of the Turkish context, while a deliberate and theoretically productive choice, means that the applicability of the findings to other cultural settings remains an empirical question. Notably, retrospective life story accounts are themselves a form of intergenerational transmission, as narrating one’s own story necessarily draws on experiences and meanings passed down across generations [Thompson, 1997, quoted in Brannen (2015)], lending methodological coherence to the retrospective self-report design employed here. Lastly, although the sample was experientially homogeneous, it varied along demographic dimensions such as occupation, child age, and marital status; future IPA studies might profitably examine more demographically bounded subgroups (for example, fathers of young children only) to surface experiential nuances that the present breadth may have smoothed over.

### Future directions

In light of the findings of this study, it is of critical importance that future research be conducted with more heterogeneous samples encompassing fathers from different socio-economic and cultural groups to understand how the interaction between trauma and masculinity operates in different contexts. Methodologically, it is recommended that observational methods be used to support self-reported data and that longitudinal designs be prioritized to track the persistence of transformations in the fathering process over time. The other core point, however, is the development and testing of the effectiveness of culturally and trauma-sensitive intervention programs for fathers with a history of trauma; such clinical support and psychoeducational initiatives will play a fundamental role in preventing the intergenerational transmission of trauma and in building healthier parenting models.

## Data Availability

The raw data supporting the conclusions of this article will be made available by the authors, without undue reservation.
