# Correction: Reconstructing fatherhood after childhood trauma: a phenomenological study of Turkish men

**DOI:** 10.3389/fpsyg.2026.1947366

**Published:** 2026-08-18

**Authors:** Özge Erduran Tekin, Eda Ermagan Caglar, Cagla Sahin

**Affiliations:** 1 Department of Educational Sciences, Turkish Air Force Academy, National Defense University, Istanbul, Türkiye; 2 Department of Psychology, Faculty of Humanities and Social Sciences, Sakarya University, Sakarya, Türkiye; 3 Institute of Social Sciences, Uskudar University, Istanbul, Türkiye

**Keywords:** childhood trauma, fatherhood, intergenerational trauma, interpretative phenomenological analysis, masculinity norms, parenting experiences

In the published article, there was an error in the affiliation for Author Özge Erduran Tekin. As published, the affiliation was incorrectly given as “Department of Humanities and Social Sciences, Air Force Academy, National Defense University, Istanbul, Türkiye.”

The correct affiliation is “Department of Educational Sciences, Turkish Air Force Academy, National Defense University, Istanbul, Türkiye.”

The original article has been updated.

